# Verdict of a Coroner’s Jury

**Published:** 1885-02

**Authors:** 


### VERDICT OF A CORONER'S JURY.

"Gus McAfee, a negro, aged about 28 years, died suddenly yesterday at his home on Pine Street. Coroner Hilburn held an inquest and the jury returned a verdict of death from congestion."

The above notice appeared in the *Constitution* of the 4th of January, and seems to dispose of a common place matter in a very satisfactory manner, and may lead some to suppose that there had been "much ado about nothing."

This result of rather elaborate proceedings, with an autopsic exploration, made in due form by competent members of the medical profession, reminds us forcibly of the pulling and hauling of a patient by a dentist who had been engaged to extract a tooth at a stipulated price, much below the ordinary rates for such service. After he had dragged the close-fisted owner of the aching tooth about the room and was almost upsetting the chair by wrenching his head from side to side, a parley was called for; and the subject demanded an explanation of what he meant by inflicting such suffering without extracting the tooth, when he replied that he was taking a quarter of a dollar pull at his tooth. The interlocutor wished then to know if it would make any more progress or lessen the difficulty about coming out, should he pay more. Our dentist told him that if he paid a dollar in advance the pullicans might be relied upon to do their work promptly, and accordingly upon entering with the cash the extraction was effected forthwith. But the reader is at a loss to see the application of this well paid, expeditious, and effective dental operation to the case of cutting open the body of a miserable wretch who is supposed to have died from congestion, as stated in the verdict of a coroner's jury; because forsooth the county physician did not think proper to submit the contents of the stomach to an analysis or to subject the brain to proper exploration, lest the commissioners might not grant any indemnity for such services.

We are prepared to resolve this twenty-five cents pull at the bowels and other viscera of the negro, with its result, by assuring all concerned that a clearing up of the doubts raised in regard to the cause of death in this case could only have been satisfactorily disposed of by instituting such examination as was suggested to the county physician on the occasion, but rejected on account of the pecuniary cost of such investigation; and that in the absence of such exploration, this general term, which indicates nothing more than the word hypertrophy as applied to disorders of the system, was a happy expedient for the emergency, so that congestion was brought into requisition to cover up any definite knowledge of the real cause of the convulsions which preceded the death of this young colored man.

It is not expected that a coroner's jury shall be empaneled, and that the body of a corpse shall be opened for inspection, upon the declaration of the attending physician that he is unable to declare the cause of death, without a reasonable presumption of foul play in the fatal result; and that any proper investigation should be set aside by the officials, from the impression that some expense to the State might ensue, is, to say the least of it, a quarter of a dollar pull at the cause of trouble. Either abstain entirely from all inquiry into such cases, or let the investigation be conducted in a way to get at the truth, even should it involve some pecuniary outlay to the Commonwealth of Georgia. Every citizen is interested in the faithful execution of the law in cases of sudden death. The fact being known that in all suspected cases, or in which there is a reasonable doubt as to the cause of death, a thorough and proper examination shall be made of everything calculated to throw light upon the fatal result, is the only guarantee of the community against the use of secret means for carrying out the vindictiveness of those who may be so inclined. The greatest probability of fixing the act upon the evil doer is established by the certainty of poison being taken without the knowledge of the person receiving it, and no trouble or expense should be spared in tracing out all the details connected with a death which is not clearly attributable to the ordinary operation of disease. This slip-shod verdict of "death from congestion" by the coroner's jury, is calculated to mislead the public, as it fails in all the essential requisites of a proper indication in respect to the fatal result, and it is held that methodic investigation should have given correct information of the facts in the case.

